# Void Swelling Induced Surface Modifications: Exploring the Relation between the Crystallographic Orientation and Surface Facets

**DOI:** 10.1002/smsc.202500172

**Published:** 2026-01-13

**Authors:** Selvaraj Julie, Christopher David

**Affiliations:** ^1^ Indira Gandhi Centre for Atomic Research A CI of Homi Bhabha National Institute (HBNI) Kalpakkam Tamilnadu 603102 India; ^2^ Materials Science Group Indira Gandhi Centre for Atomic Research Kalpakkam Tamilnadu 603102 India

**Keywords:** ion irradiations, molten salt reactors, nanocrystalline nickel, surface facets, void swelling

## Abstract

The phenomenon of surface facet formation during ion implantation has captured considerable scientific and technological interest. Surface facets—including wavy, pyramidal, and terraced morphologies—are typically formed during off‐normal keV and MeV ion beam implantation, and due to injected gas effects. In certain circumstances, these features may also emerge during irradiation at normal incidence: when differential sputtering occurs in biphasic regions, when contaminants are inadvertently added as dopants, or when the experimental arrangement permits the coimplantation of metals. The formation of surface nanopatterns in nanocrystalline nickel under high‐temperature ion irradiation at normal incidence has been observed—a phenomenon that conventional mechanisms fail to explain. A novel mechanism driving nanopattern formation under these conditions is presented. These findings offer compelling evidence that facets result from voids forming on the surface and in its vicinity. A strong correlation between the crystallographic orientation and the facet type has also been identified. Specifically, grains oriented in the <100> and <111> directions display smooth and wavy morphologies, while grains with orientations in between exhibit more complex shapes. The research indicates that grains with low stress and surface energies tend to exhibit wavy facets, while higher values lead to the formation of more complex shapes.

## Graphical abstract

A novel mechanism for nanopattern formation in nanocrystalline nickel under high‐temperature ion irradiation is unveiled. Facets evolve from surface and subsurface voids, with their morphology dictated by crystallographic orientation. The <100> and <111> grains develop smooth or wavy facets, while others exhibit intricate designs. Low stress and surface energies favor wavy facets, whereas higher values result in more complex shapes.

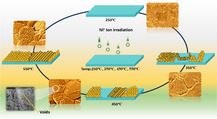

## 1 Introduction

Gen‐IV reactors, like molten salt reactors (MSRs), offer a path to carbon‐free electricity generation while prioritizing safety and security. One of the main challenges in the near‐term deployment of MSRs is the corrosion of structural materials in the primary and secondary reactor loops due to the molten fluoride salts (FLiNaK). Recent research has shown that nanocrystalline (NC) Ni coating enhances the resistance of structural materials to corrosion and intergranular cracking compared to uncoated materials.^[^ ^1^ ^]^ The attributes of the coatings, such as grain size, texture, grain boundary characteristics, and surface roughness, influence their corrosion behavior.^[^ ^2^, ^3^, ^4^, ^5^, ^6^ ^]^ Neutron and ion irradiation in various materials have shown that displacement damage affects these attributes.^[^ ^7^, ^8^, ^9^, ^10^, ^11^, ^12^ ^]^ This emphasizes the importance of studying how these characteristics in NC‐Ni respond to irradiation.

If NC‐Ni is used for coating the structural materials of MSRs, apart from the corrosive medium of the fuel carrier, they would be exposed to fission neutrons, fission fragments having energies of ≈1 MeV amu^−1^ and transmutation products, which include helium. Considering the impact of surface roughening on corrosion behavior, various potential mechanisms by which these energetic particles (the fission neutrons, fission fragments) and helium atoms lead to roughening in NC‐Ni become important. Curvature‐dependent sputtering is important for roughening and nanopatterning of solid surfaces; this is well known for low‐energy ion irradiations^[^ ^11^ ^]^ and, to a lesser extent, at higher energies.^[^ ^13^ ^]^ The sputtering caused by neutrons of various energies has been investigated,^[^ ^14^ ^]^ but it is orders of magnitude smaller than that caused by ions in the keV range, which undermines their role in surface modifications. Fission fragments have electronic stopping power similar to swift heavy ions with energies of a few MeV amu^−1^ and therefore, cause surface erosion through electronic sputtering.^[^ ^15^ ^]^ The sputtering caused by electronic excitations exceeds that induced by elastic collision cascades during keV ion bombardment.^[^ ^15^, ^16^ ^]^ Therefore, a significant effect of fission fragments on surface roughening is anticipated. Besides, helium‐induced surface morphologies, such as blisters,^[^ ^17^ ^]^ pores,^[^ ^18^ ^]^ ripples,^[^ ^19^ ^]^ and grass structures,^[^ ^18^ ^]^ are formed due to the helium bubbles exerting pressure on the surface. Therefore, helium produced during transmutation reactions in nickel could cause surface morphologies in NC‐Ni. The radiation‐induced phenomena in MSRs resulting from neutron irradiation and fission fragments can be studied through heavy ion implantation with suitable energies^[^ ^20^, ^21^ ^]^ and gaseous ion implantations for transmutation gas effects.^[^ ^22^ ^]^

In this study, we investigate the formation of surface patterns in NC‐Ni caused by MeV ion irradiation at normal incidences within the temperature range of 250–550 °C. The production of surface morphologies in metallic targets using MeV ion irradiation has been recently demonstrated.^[^ ^13^ ^]^ The studies observed ripple formation at incident angles beyond a critical angle of ≈45°, resembling the formation in semiconducting materials subjected to low‐energy ion irradiations.^[^ ^11^ ^]^ The continuum models revealed the interplay of mass redistribution, curvature‐dependent sputtering, and ion implantation as the physical mechanism governing the ripple formation and also closely predict the critical angle. Based on current consensus, 1) ripple formation starts at nonzero critical angles irrespective of the substrate type, that is, metals, semiconductor or insulator^[^ ^13^ ^]^ and 2) pattern formation below critical angles and at normal incidences as demonstrated in silicon, is the result of unintentional codeposition of impurities or nanopatterning achieved in specific experimental arrangements that enable codeposition of metals during ion beam sputtering.^[^ ^23^ ^]^ A recent study observed a rippled structure in nickel during high‐dose helium implantation at normal incidence, attributing to high electronic energy loss effects.^[^ ^19^ ^]^ Additionally, surface manifestations can occur when metals are exposed to swift heavy ion irradiation with very high electronic stopping power.^[^ ^24^ ^]^

Given the extensive reporting on the formation of ripples, the emergence of nanoscale surface patterns under normal incidence and nongaseous ion irradiations used in the present study is intriguing. Here, we report a novel mechanism for the formation of surface facets in NC‐Ni during heavy ion irradiations. NC‐Ni is irradiated with 1.4 MeV nickel ions in the temperature range of 250–550 °C at normal incidence. Scanning electron microscopy (SEM) observed facets of various types and smooth surfaces, which were strongly correlated to the grain orientation deduced from electron beam backscatter diffraction (EBSD) measurements. Through transmission electron microscopy (TEM), compelling evidence is established that gas‐atoms‐free voids produced at and near the surface are responsible for developing facets with dimensions in the nanometer range. We demonstrate that varying levels of stress and surface energies in grains of different orientations lead to the formation of different facets. Notably, we found that low values of both produce wavy facets, while higher values result in the development of more complex shapes.

## 2 Results

### 2.1 Surface Modification by Ion Beam Irradiation

**Figure 1:**
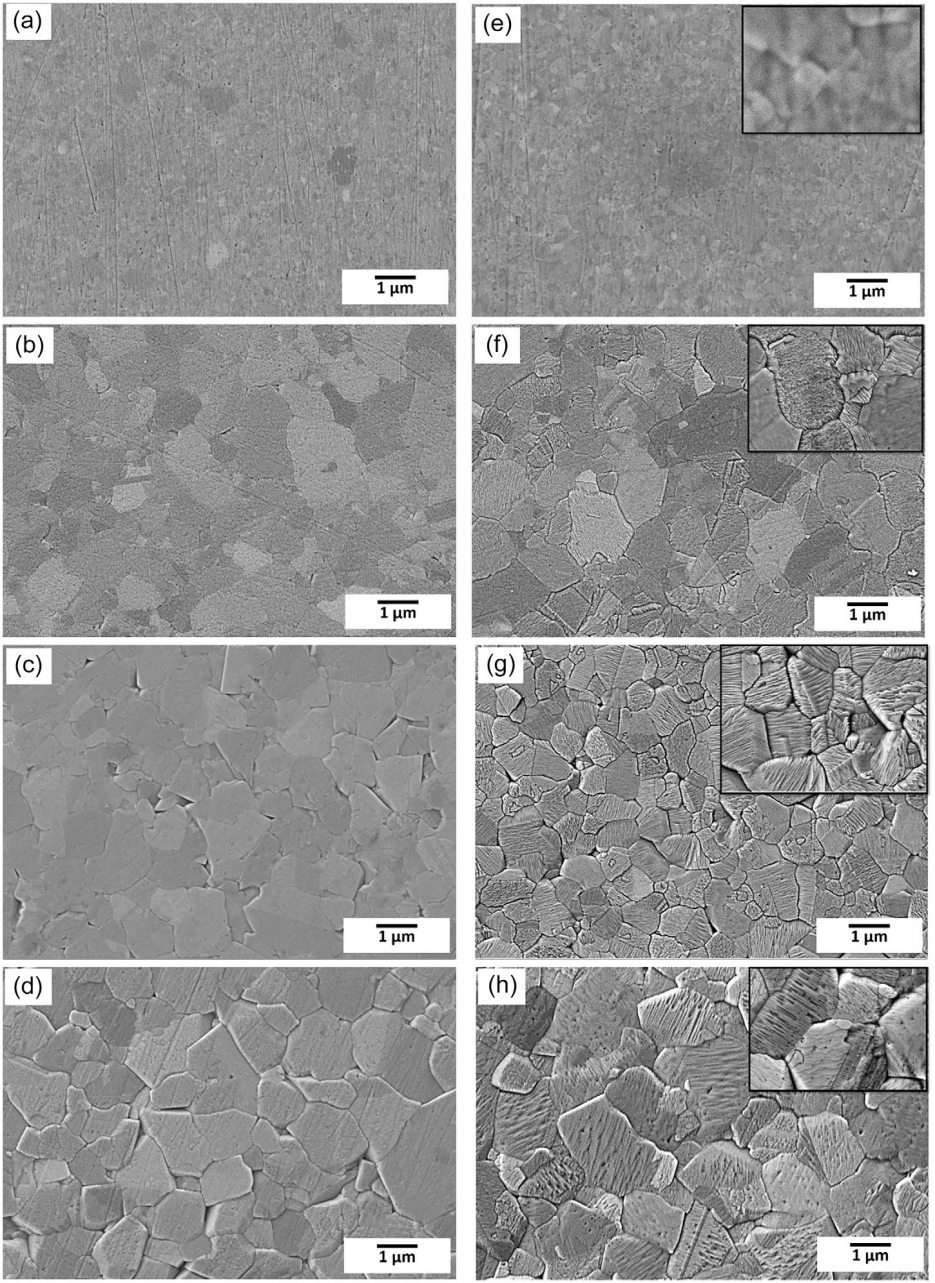
SEM image of unirradiated and irradiated areas of NC‐Ni sample at 250, 350, 450, and 550 °C. a,b,c, and d) represents the unirradiated areas and e,f,g, and h) the corresponding irradiated areas at 250, 350, 450 and 550 °C, respectively. Irradiation‐induced faceted surfaces are visible in (f,g, and h).

The NC‐Ni samples in the as‐deposited state have an average grain size of 20 nm; these were irradiated with 1.4 MeV Ni^+^ ions up to 18.5 dpa (displacements per atom) at various temperatures (250, 350, 450, and 550 °C). The surface morphology of unirradiated and irradiated areas of NC‐Ni samples at 250, 350, 450, and 550 °C is shown in **Figure** **1**. It can be seen that the microstructure of NC‐Ni evolves with temperature in both unirradiated and irradiated areas. Grain growth in the unirradiated and irradiated areas from the as‐deposited NC state can be observed, which is due to high grain boundary and strain energies in the as‐deposited NC‐Ni. Grain growth occurred at all temperatures, and the growth rate increases with temperature. Consequently, the microstructure is not uniform throughout this temperature range. In irradiated areas, ion irradiation further accelerates these thermally driven processes, as detailed in our previous studies.^[^ ^10^ ^]^ The morphological dissimilarities between unirradiated and irradiated surfaces are noticeable at all temperatures but become more prominent at higher temperatures. The irradiated areas show rumpled surfaces, known as facets, whereas these features are absent in the unirradiated areas (Figure 1). Faceted surfaces are formed during irradiation at temperatures of 350–550 °C (insets to Figure 1f, g, and h). The different facet morphologies arising during irradiation are given in Figure S1–S3 (Supporting Information). The inset to Figure 1e shows that these features are absent on surfaces irradiated at 250 °C. The lack of surface facets is emphasized in a panoramic SEM image of the surface irradiated at 250 °C (Figure S4, Supporting Information). The atomic force microscopy (AFM) images comparing the surface morphology of samples irradiated at 250 °C with those of as‐deposited (unirradiated) samples and samples irradiated at 350 °C (see Figure S5, Supporting Information) further demonstrate that the surfaces irradiated at 250 °C do not exhibit any facets.

There are reports of the development of surface morphology, such as facet formation and growth during thermal annealing.^[^ ^25^, ^26^ ^]^ These facets are usually formed at high temperatures over extended annealing periods. Besides, the surface reconstruction during high‐temperature oxidation^[^ ^27^ ^]^ and recrystallization‐induced roughening leading to changes in the surface morphology is reported.^[^ ^28^ ^]^ Our earlier study showed that the recrystallization temperature of the NC‐Ni sample is 450 °C.^[^ ^10^ ^]^ Here, all irradiations were carried out in ultra‐high vacuum (UHV) conditions where surface oxygen contamination is expected to be minimal. Hence, the absence of surface facets in the unirradiated area at 450 °C (Figure 1c) suggests that recrystallization‐induced roughening and surface oxidation do not affect surface morphology change. To investigate the effect of annealing temperature and duration on surface morphology, the NC‐Ni sample was annealed at 650 °C for 24 h. The SEM image of the Ni sample that underwent long‐duration annealing shows smooth grains devoid of surface morphological features (Figure S6, Supporting Information). Therefore, it appears that facet formation through annealing alone would require much higher temperatures. In addition, the reported annealing‐induced facets are in micron range roughness,^[^ ^25^, ^26^ ^]^ whereas in the present case, surface facets in the irradiated areas vary from 5 to 20 nm (Figure S7, Supporting Information). It emphasizes that surface facets formed in the irradiated areas are the effects of the Ni ion irradiation.

Furthermore, mechanical polishing introduces surface plastic deformation, but such stresses are relieved upon annealing. It can be seen from Figure 1 that polishing scratches are visible in the unirradiated areas, particularly in both the unirradiated and irradiated areas at the lowest temperature (250 °C) (Figure 1a and e). If residual mechanical stresses are responsible, facets would appear in all unirradiated areas, which is not the case. Similarly, if such stresses influenced the irradiation process, facets would be most prominent at 250 °C, where the stress relief during annealing is minimal; however, no facets are observed in the irradiated regions at this temperature. This clearly demonstrates that facet formation is solely driven by irradiation and not influenced by residual mechanical stress. A recent investigation reports the development of surface morphologies initiated by preferential sputtering in biphasic regions of hypereutectic alloys by ion irradiations at normal incidence.^[^ ^29^ ^]^ In the present experiments, if facet formation were due to sputtering, it should have occurred at all temperatures. The absence of facets in the 250 °C irradiations suggests that sputtering is not responsible for the facet formation.

Another study observed a rippled structure in nickel during high‐dose helium implantation at normal incidence at room temperature.^[^ ^19^ ^]^ The rippled structure was ascribed to high electronic energy loss‐induced compressive stress. The electronic stopping power for the present irradiation conditions is $\frac{dE}{dx}=1.4{\text{ keV nm}}^{-1}$, and of a similar order to that reported $\frac{dE}{dx}=0.7{\text{ keV nm}}^{-1}$ during the helium irradiations. However, the lack of surface manifestations at 250 °C and their occurrences only at higher temperatures suggest a different underlying mechanism. The stress induced by irradiation, which is dependent on the concentration of the implanted ions and point defects, promotes ripple growth.^[^ ^30^ ^]^ Our previous study^[^ ^10^ ^]^ showed that the residual vacancy concentration is highest in NC‐Ni irradiated at 250 °C; hence, stress‐induced ripples are expected to form at this temperature, which is not the case. Hence, stress plays a less significant role in ripple formation under these irradiation conditions. If irradiation was the sole contributing factor to pattern formation, then the 250 °C sample should have displayed a faceted surface since the damage rate is identical for all temperatures. The smooth surface encountered during the 250 °C irradiation implies that the irradiation temperature is also an essential factor for facet formation. Based on the above observations, the surface morphological changes that occurred in the irradiated areas are due to the combined effects of the heavy ion irradiation and irradiation temperature.

As evident from Figure 1 and Figure S1–S3 (Supporting Information), the irradiation‐induced facet formation, which is observable at 350 °C, becomes significant at 450 and 550 °C. Based on their appearance, the facets on the irradiated surface are categorized into wavy, pyramids, terraces, and needle‐like morphologies (see Figure S1–S3, Supporting Information), and those grains devoid of facets are referred to as smooth morphologies. In addition, other morphologies are also observed in the irradiated areas. Since the other morphologies are the subset of the primary morphology, differing by their wavelength or number of steps in the pattern, the present work deals only with the aforementioned morphologies.

**Figure 2:**
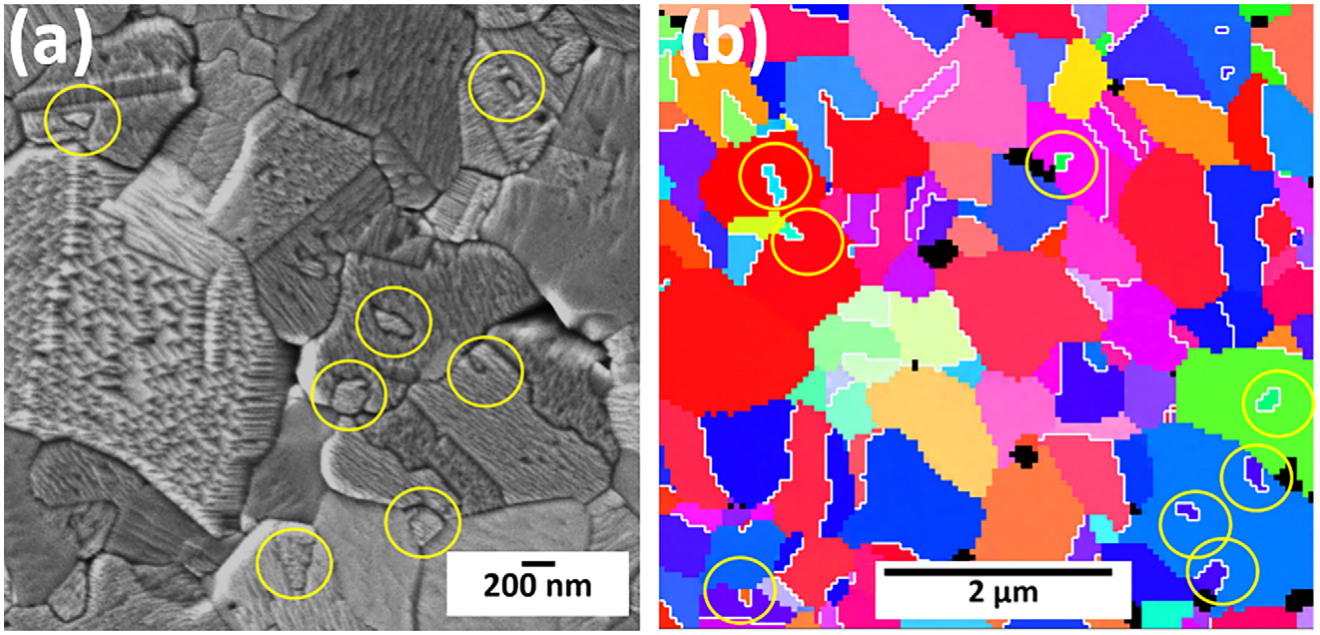
Island grains differing in facet morphology compared to matrix grains: a) The SEM image shows the island grains formed in the irradiated area at 450 °C, which are marked with yellow circles. The island grains have different facet morphology compared to the matrix grain; b) the EBSD IPF‐Z map shows the island grains have different orientations; the white lines represent the Σ3 CSL twin boundaries. It should be noted that SEM image and EBSD map are taken from different sample locations.

Furthermore, several island grains (marked with yellow circles in **Figure** **2**a), which are relatively smaller compared to the average size of the grains, are embedded within the grain. Figure 2b shows the EBSD inverse pole figure‐Z (IPF‐Z) orientation image and white lines represent the Σ3 coincidence site lattice (CSL) twin boundaries. It can be seen that the island grains are surrounded by Σ3 CSL twin boundaries. Σ3 twin boundaries have low interface energy and higher atomic ordering. The Σ3 CSL relation associated with the island grains results in lower mobility of grain boundaries (GBs); therefore, these boundaries migrate at low velocity during high‐temperature annealing or irradiation. This is the reason for island grain formation. Additionally, the island grains in the irradiated areas showed different facet geometry compared to its matrix grain (Figure 2a). This difference in morphology arises from the differing crystallographic orientations of the island grain and the matrix grain (Figure 2b).

### 2.2 Orientation of Facets

**Figure 3:**
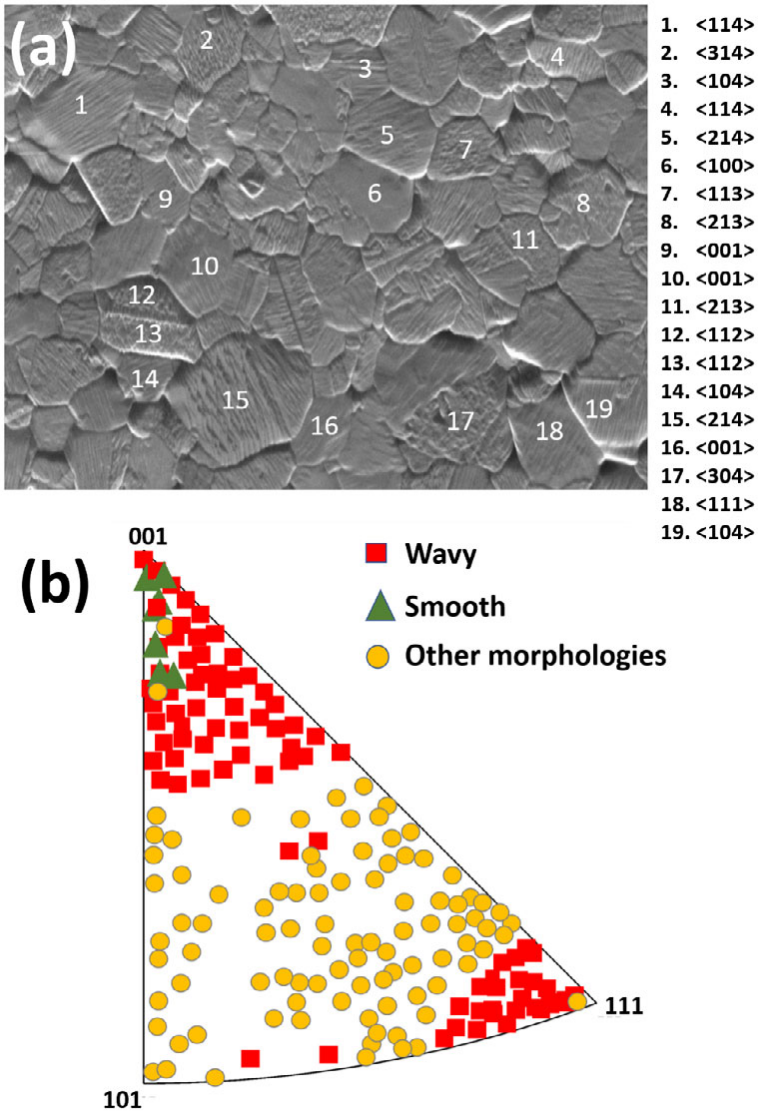
Orientation‐dependent facet formation: a) EBSD electron image of facet morphologies in the irradiated area at 450 °C, the grains with different morphologies are numbered and the corresponding orientations are given alongside; b) these orientations are plotted in inverse pole figures. Over 100 grains from various irradiated areas are analyzed to determine the orientation dependence of facets, and the corresponding orientations are also plotted in (b).

In order to ascertain the relation between surface facets and crystallographic orientations, EBSD orientation maps are taken from different surface morphologies of grains in the irradiated areas. At 350 °C, the facet morphologies are the same as those at 450 and 550 °C, but significant portions of the surface are smooth, that is, facet‐free. In the case of 450 °C, only a small fraction of the grains are smooth, whereas at 550 °C, the entire grain surfaces are faceted (Figure 1). Since surface facet types are analogous at all temperatures except at 250 °C, the orientation dependence of facet formation analysis was carried out only at 450 °C. A typical analysis of an irradiated region is shown in **Figure** **3**a. Grains containing various surface morphologies, such as wavy, pyramids, smooths, steps, terraces, etc., are numbered in the figure. The corresponding grain orientations given alongside are plotted in the inverse pole figure (Figure 3b). The facet morphologies, such as pyramids, steps, terraces, and other complex shapes, are grouped into a single “other morphology” category. A similar analysis was conducted over 100 grains of various irradiated areas to ascertain the orientation dependence of facets; the corresponding orientations are also presented in Figure 3b. It can be seen that grains with orientations close to <100> with a deviation of 5° exhibit smooth morphology, while those with a deviation of 20° develop wavy morphologies, whereas grains close to <111> with a deviation of 10° tend to develop wavy morphologies. Grains with other orientations between <100> and <111> are more likely to form different morphologies.

### 2.3 Origin of Unique Surface Features by Irradiation

**Figure 4:**
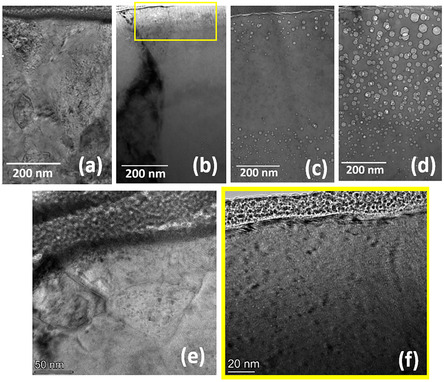
Void distribution at different irradiation temperatures. The cross‐sectional bright‐field‐TEM (BF‐TEM) images of NC‐Ni irradiated with 1.4 MeV Ni^+^ ions to a dose of 1.3 × 10^16^ ions cm^−2^ at various temperatures a) 250 °C, b) 350 °C, c) 450 °C, and d) 550 °C. The voids are observed just below or on the surfaces except 250 °C. The higher magnified images of NC‐Ni irradiated at e) 250 °C and f) 350 °C are shown. (f) A magnified image of the region within the yellow rectangular box clearly shows the presence of voids near to the surface. BF images were acquired with a defocus value of ±1.6 μm.

**Figure 5:**
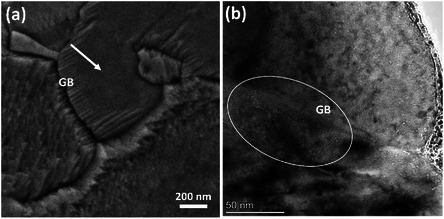
Relation between voids located at GBs and facets in their proximity: a) SEM image of NC‐Ni irradiated at 350 °C, showing facets near the GBs. No facets are present inside the grain, as indicated by the white arrow. b) Cross‐sectional TEM image showing a high density of voids near the GBs (marked by the white circle) compared to the grain interior.

To determine the origin of facet formation, the cross‐sectional TEM was carried out in the irradiated areas at all temperatures. The TEM cross‐sections at 250, 350, 450, and 550 °C are shown in **Figure** **4**. Voids, which are present at all temperatures except 250 °C, occur throughout the implanted range. The voids are smaller at 350 and 450 °C, whereas at 550 °C, the void size is significant, up to 50 nm. The depth profiles of void density distribution and swelling for 350–550 °C are given in Figure S8 (Supporting Information). It can be observed that there is a significant decrease in void density between 200 and 400 nm at 450 °C, whereas at 550 °C, a slight decrease in this range. This is because of the injected interstitial effect, which is not present at 350 °C. The details of void distribution at these temperatures are discussed in our recent work.^[^ ^31^ ^]^ It has been suggested that a void‐denuded zone develops at the surface during irradiation due to the surface effects, acting as a strong sink for point defects and preventing void nucleation.^[^ ^32^ ^]^ However, the absence of a surface void‐denuded zone at various temperatures (350–550 °C) in Figure 4 is attributed to strong internal sinks (dislocations, GBs, and voids), hindering the movement of point defects toward the surface. Higher dislocation densities (≈10^15^ m^−2^) at the onset of irradiation^[^ ^33^ ^]^ are expected to significantly decrease the widths of void‐denuded zones.^[^ ^31^ ^]^ Additionally, higher dpa rates at the surface (≈10 dpa) lead to a surplus of vacancies and interstitials, in which interstitials are lost to dislocations and surfaces, whereas vacancies aggregate to form voids, preventing void denudation in the surface and adjacent regions across all temperatures.^[^ ^31^ ^]^ Thus, voids are the consequence of the faceted structures on the surface. This is the first report that irradiation‐induced voids are the source of facet formation. In addition, **Figure** **5**a shows the faceted structures formed near the GB at 350 °C, but these features are absent in the grain interior and appear smooth. The cross‐sectional TEM image in Figure 5b clearly shows the higher density of voids near the GBs than in the grain interior, providing further compelling evidence that voids are the cause of the observed facets.

### 2.4 Relation between Surface Facets and Voids at the Surface

**Figure 6:**
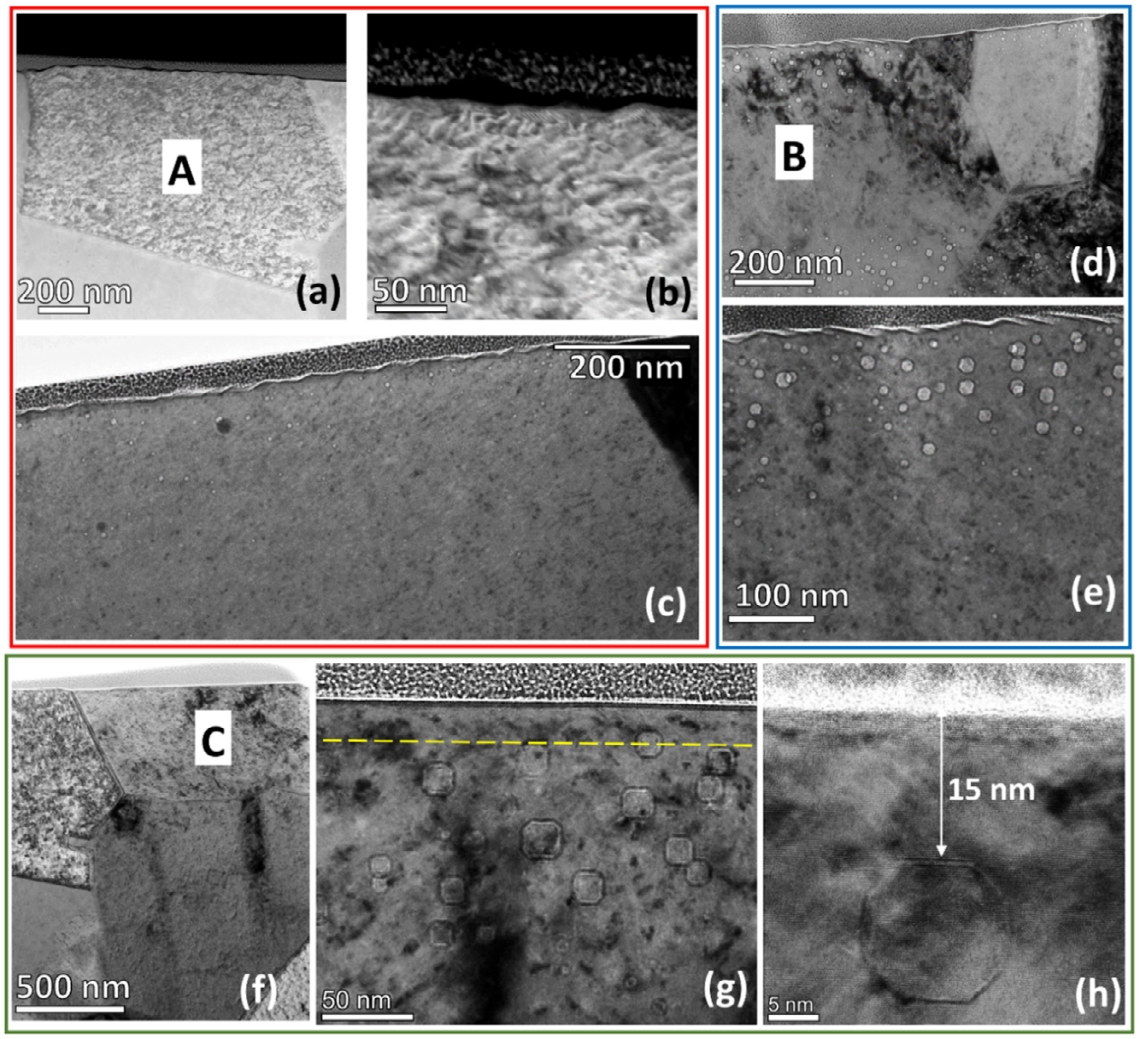
Importance of surface voids in the formation of facets in NC‐Ni irradiated at 450 °C: a) high‐angle annular dark‐field scanning transmission electron microscopy (HAADF‐STEM) image of the faceted surface in “A” grain, b) the corresponding high magnification image clearly depicting the facets, and c) corresponding BF‐TEM image showing the presence voids underneath the faceted surface; d) BF‐TEM image of “B” grain showing faceted surface and e) corresponding higher magnification image clearly shows the voids underneath the faceted surface; f) HAADF‐STEM image of “C” grain showing the smooth surface, g) corresponding BF‐TEM image showing the smooth surface and voids that are present away from the surface indicated by yellow dotted lines, and h) High‐resolution TEM image of a void present in “C” grain at a distance of 15 nm away from the surface.

To understand the relation between the surface facets and voids at the surface, TEM analysis is performed on two faceted grains, “A” and “B” and a smooth grain, “C” (**Figure** **6**). The grain “A” is close to <111>//ED and the smooth grain “C” is in the <100>//ED orientation. It can be seen that in grain “A” where the voids are present in close proximity to the surface (≈1–2 nm from the surface), surface undulations can be observed (Figure 6b and c). Similar observations can be made in grain “B” (Figure 6d and e). In contrast, voids distant from the surface (see the void‐denuded zone width of 15 nm in Figure 6g and h) cause only smooth surface morphology. These observations provide compulsive evidence that surface voids are responsible for the formation of surface facets.

**Figure 7:**
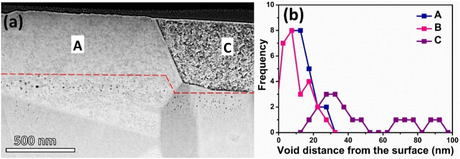
The crucial role of void distances from surfaces for facet formation: a) HAADF‐STEM image of “A” and “C” grains, the voids appear in black and are discernible. Notably, the voids in the “C” grain appear to be deeper in comparison to those in the “A” grain. The positions beyond which voids occur in grains “A” and “C” are represented by a red dashed line. The grains “A” and “C” referred here are the same as those shown in Figure 6. b) The void distribution in the top 100 nm from the surface in grains “A”, “B”, and “C”. The grains “A”, “B”, and “C” mentioned here are the same as those in Figure 6.

As discussed in the earlier section, if a high fraction of dislocation density was the cause of the lack of surface void‐denuded zone, then what is the cause of surface void denudation in certain grains, such as grain “C” (Figure 6g and h)? **Figure** **7** displays high‐angle annular dark‐field scanning transmission electron microscopy (HAADF‐STEM) of the grains, “A” and “C”, of which facets in “A” resulting from surface voids can be observed; on the other hand, the lack of them on “C” resulted in their smooth surfaces. The position corresponding to a deeper layer of void formation in either grain is indicated by a red line in Figure 7a. It is observed that voids are found deeper in grain “C”, which is <100> oriented, than in grain “A” of <111> orientations. Additionally, the void distance from the surface is plotted for A, B, and C grains in Figure 7b. In both A and B grains, voids are present on the surface and nearby; however, in “C”, the void distance from the surface varies from 15 to 100 nm.

To elucidate these observations, it is essential to explore the channeling effects of incident ions in grains with <100> and <111> orientations. At ion energies used in the experiment, both nuclear and electronic stopping mechanisms contribute to the overall stopping power. For channeled ions, a high screened Coulombic potential due to atoms in the string and low atom density within the channel reduces interactions of incident ions with atomic nuclei and electrons of the target. In axial channeling, the potential is inversely related to interatomic distance, while atom density within the channel exhibits a direct relationship. As observed in silicon, these reduce energy loss in the <110> direction compared to <100> due to a smaller interatomic distance along the former.^[^ ^34^ ^]^ In the present case, the interatomic distance along <100> (0.352 nm) is smaller than <111> (0.609 nm) orientations. Therefore, the stopping power of ions is expected to be lesser along <100> than <111>.

Besides, the depth at which dechanneling occurs, that is, the dechanneling length, is more significant in channels with higher atomic potential and low atomic densities within the channel. The concept of critical angle is well known in ion channeling studies. Higher critical angles point to considerable transverse energies required for ions to undergo close encounters with atomic strings and larger dechanneling lengths.^[^ ^34^ ^]^ The calculation for critical angles for the present experimental conditions is given in the Supporting Information. Calculations show <100> has a greater critical angle than <111>, implying higher dechanneling lengths for <100>. Therefore, the smaller energy loss and higher dechanneling length for ions incident along <100> are reasons why void formation at both near‐surface and deeper regions occurs at greater depths in grain “C” compared to grain “A”.

At a temperature of 550 °C, the facets on the surface are clearly visible in the image shown in Figure S9 (Supporting Information). Furthermore, surface voids across all grains imply increased dechanneling of incident ions, primarily attributed to increased thermal vibrations of atoms. In Figure S9 (a–c) (Supporting Information), even though voids appear close to the surface; however, a slightly smoother surface morphology is observed. This is due to the coarsening of the facets that result from increased surface diffusion, which is expected at higher temperatures.

## 3 Discussions

The research indicates that the areas exposed to irradiation exhibit facets that result from the simultaneous impact of irradiation and elevated temperatures. Our findings have significant implications, showing that surface voids play a crucial role in facet development, and the type of facets is intricately linked to crystallographic orientations. It is well‐known that the limited depth undergoing swelling during ion irradiation is laterally constrained by the unirradiated substrate. In the present study, the irradiated layer, which undergoes swelling, accounts for only 0.2% of the overall specimen thickness and the lower/deeper regions of the swelling volume are constrained by shear components at the unirradiated interface. Due to the rigidity of the unirradiated area, compressive stress is generated, and irradiation creep operates concomitantly to relieve this swelling‐generated stress. Therefore, there is a mass transport toward the incident ion beam direction by dislocation motion. Since the creep rate is orientation‐dependent,^[^ ^35^ ^]^ different levels of stress are encountered in differently oriented grains, and variations in surface grain extrusions are expected.

### 3.1 Mechanism for Surface Facet Formation

As discussed, the creep process acting to relieve the stress generated by void swelling causes mass transport toward the incident ion beam direction. A minimal swelling of less than 0.1% is sufficient to trigger the creep process.^[^ ^36^ ^]^ Since void swelling is generally expected to follow the damage profile, time dependence of stress can be expected, with the stress development at the peak damage preceding successive evolution at positions of decreasing damage rate. Although swelling is generally expected to correlate with the local radiation dose, several experimental studies reveal enhanced swelling near the surface and reduced swelling in regions corresponding to peak damage. The void swelling depth profiles for NC‐Ni samples irradiated at 350–550 °C (see Figure S8, Supporting Information) display “M"‐shaped patterns, featuring one peak near the surface and another beyond the peak damage zone. Recent experiments and modeling studies on self‐ion irradiated Fe, by Shao et. al., suggest that such profile shapes result from the sensitivity of void nucleation to the spatial separation between excess vacancies and interstitial defects generated during ion irradiation.^[^ ^37^ ^]^ The excess defect profile is marked by a surplus of vacancies near the surface, decreasing with depth before increasing again around half of the projected ion range. Near the projected range of the incident ions, there is a distinct excess of interstitials. Considering this defect profile, at the early stages of irradiation, the excess vacancies at the surface may lead to initial void nucleation, with subsequent void formation occurring at the second vacancy excess region as irradiation progresses. The defect imbalance is most pronounced when defect interactions are dominated by recombination, leading to a loss of vacancies available for void nucleation. The modeling results show a progression from surface‐dominated swelling to M‐shaped swelling, eventually transitioning to swelling at depths associated with the deeper excess vacancy defects as the dose rate decreases.^[^ ^37^ ^]^ The former effect is particularly prominent at high dpa rates, resulting in surface‐dominated swelling. At low dpa rates, where most interstitials are annihilated at sinks and the vacancy excesses at the deeper regions remain largely undisturbed, swelling occurs forming a deep peak. Intermediate dose rates result in the development of void swelling in deeper regions, alongside the more readily formed surface swelling. The dpa rates in our experiments are slightly smaller than the dpa rates in Shao et. al., experiments (6 × 10^−3^), therefore, the presence of “M” shaped void swelling depth profiles in our experiments is understandable.

**Figure 8:**
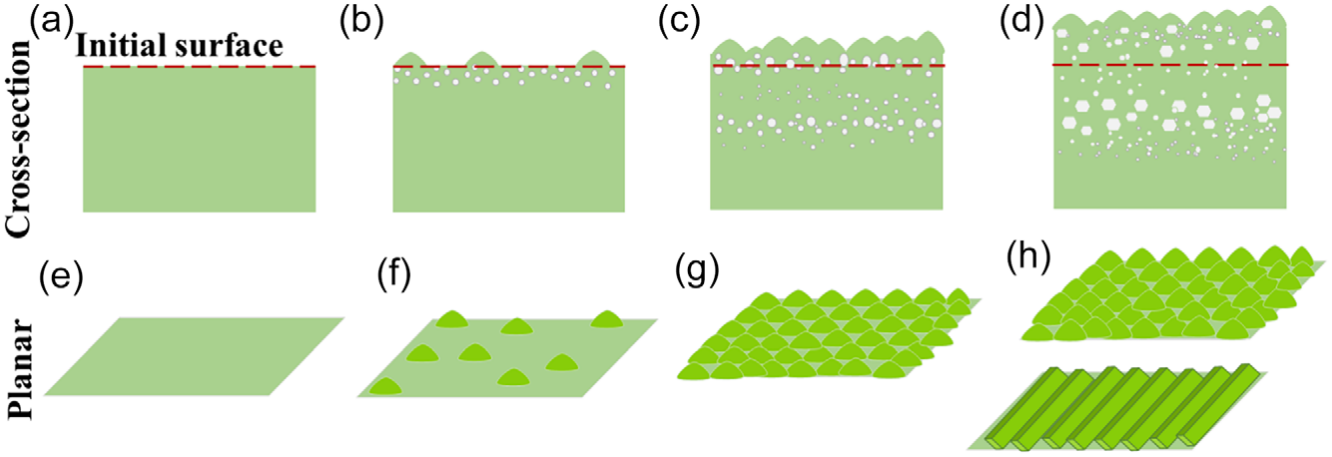
A schematic representation of the mechanism for surface facet formation during irradiation: a,e) initial surface of the NC‐Ni sample, b,f) void formation at the near‐surface regions preceding formation at depths. The swelling‐induced stress creates material flow toward the surface, forming islands. This leads to surface instability and the formation of facets. c,g) and d,h) continuing irradiation results in the formation of voids at depths, causing the material to flow to the surface, thereby increasing the surface levels and causing grain extrusions.

In an earlier study, Shao et al. traced the evolution of void depth profiles during 3.5 MeV Fe irradiations in high‐purity Fe.^[^ ^38^ ^]^ They demonstrated that as dpa increases, the void depth profiles evolve from an initial subsurface swelling for low fluences to “M"‐shaped profiles, closely following the development of excess vacancy profiles discussed above. Our experiments are conducted at lower energies, where the spatial separation between excess vacancies and interstitials is expected to be much smaller than in their irradiations. Consequently, vacancy annihilation is more likely to occur at greater depths. Thus, during the early stages of our irradiation at low fluences, voids initially form near the surface and gradually extend deeper with continued exposure. Therefore, at all temperatures (except 250 °C), the void formation and stress evolution at the surface should predate occurrences at successively deeper regions. This should cause material flow to the surface from regions between the surface and the layer with voids. Atoms reaching the surface congregate into islands, creating surface instability (**Figure** **8** (a,b and e,f)). This can be alleviated by a decrease in surface energy achieved through surface reconstruction and the formation of facets with low‐index planes. This process, followed by subsequent material flow from deeper regions along the ion range, results in extrusions while retaining the surface patterns (Figure 8 (c,d,g, and h)).

### 3.2 Emergence of Orientation‐Dependent Facet Types

It can be recalled that the wavy and smooth morphologies occurred in the grains with <111> and <100> orientations and complex facet shapes are displayed in orientations between <111> and <100>, such as <214>, <213>, <314>, <113>, etc. (Figure 3b). The lateral stress developed due to void swelling in a specific grain is influenced by its orientation in relation to the specimen surface. Wolfer and Garner proposed different irradiation creep mechanisms on the state of stress as a function of grain orientation.^[^ ^35^ ^]^ They report that the <111> and <100> orientations endure reduced lateral stress during void growth and higher stress is realized for in‐between orientations.^[^ ^35^ ^]^ The smaller stresses accommodated in the <111> and <100> orientations should have resulted in the formation of both wavy and smooth facets. It has been suggested in an earlier study that waves form in orientations with the highest areal density and the easiest slip.^[^ ^39^ ^]^ The easiest slip face in Ni is (111) and hence justifies the formation of wavy morphology in <111> grain orientations. A minimal amount of lateral stress is what caused the smooth morphology in <100> orientations. In addition, as shown, the formation of voids at distances far away from the surfaces, due to ease of channeling in <100> oriented grains, contributes to the smooth morphology of <100> orientations. The complex facet shapes are displayed in orientations between <111> and <100> (i.e., <214>, <213>, <314>, <113>, etc.), as shown from the IPF in Figure 3b. According to reports, higher stress is observed in orientations between the <100> and <111>.^[^ ^35^ ^]^ Hence, the complex facet shapes directly result from the elevated stress levels experienced in these orientations.

The <111> and <100> surfaces are atomically flat, close‐packed surfaces having low surface energies.^[^ ^40^ ^]^ The other orientations, like <214>, <213>, <314>, <113>, <210>, <320>, etc., are atomically rough surfaces and have high surface energies,^[^ ^40^ ^]^ resulting in unstable surfaces. As discussed, the facets arise due to an atom flux to the surface, resulting from swelling in the near‐surface regions. The process increases the anisotropy in surface energy. Therefore, the surface is rearranged into lower surface energy facets, that is, atomically smoother, more closely packed facets with lower energy than the initial surfaces. Therefore, the low surface energies and stress in the <100> and <111> result in the formation of surface facets of rather simple shapes (wavy), whereas the high surface energies and stress involved in the in‐between grain orientations require facets of complex shapes to minimize the surface energy.

### 3.3 Effect of Temperature on Facet Coverage

The results provide robust experimental evidence that irradiation at 250 °C did not result in any surface faceting, as demonstrated by the SEM images in Figure 1e and Figure S4, Supporting Information, and the AFM images in Figure S5, Supporting Information. The lack of surface facets during the 250 °C irradiations is due to the absence of voids at 250 °C. In contrast, irradiation at 350 °C led to the surface faceting (Figure 1f, Figure S1, and Figure S5, Supporting Information). Furthermore, the extent of surface facet coverage increased progressively with irradiation temperature: only a few grains showed facets at 350 °C (Figure S1, Supporting Information), while a significantly larger number exhibited faceting at 450 °C (Figure S2, Supporting Information), and nearly all grains exhibited faceting at 550 °C (Figure S3, Supporting Information).

As discussed, the proximity of voids to the surface plays a key role in facet formation: voids located at or near the surface promote faceting, whereas those situated deeper, even beyond 10 nm from the surface, lead to smooth surfaces. It was also shown that in <100> oriented grains, voids tend to form at greater depths compared to <111> oriented grains, resulting in smoother surfaces in the former and facetted surfaces in the latter. The reduced stopping power and greater dechanneling length in the <100> as compared <111> were shown to be the underlying reasons for this observation. Therefore, a predominance of <100> oriented grains in a sample would suggest a lower proportion of grains with faceted surfaces. Our previous study^[^ ^10^ ^]^ showed that most grains in the irradiated areas of the NC‐Ni at 350 °C exhibited a <100> texture (Figure S10, Supporting Information). This likely contributed to the lower proportion of faceted grains observed during irradiation at this temperature. Additionally, the void density is heterogeneous and observed to be high near the GBs as compared to the grain interiors (Figure 5), causing facet formation near the GBs. Moreover, the magnitude of void swelling recorded at this temperature is abysmally small, up to ≈0.07% (Figure S8, Supporting Information). All these factors play a role in the reduced surface coverage of facets in the 350 °C irradiated samples.

Our previous work^[^ ^10^ ^]^ demonstrates that irradiation at 450 °C enhances the thermal texture, resulting in a decrease in <100> and an increase in <111> orientations due to the radiation‐enhanced diffusion process, compared to irradiation at 350 °C (Figure S10, Supporting Information). The increase in <111> texture, combined with the higher dechanneling probability at elevated temperatures, leads to a reduced fraction of incident ions undergoing channeling. Moreover, higher surface swelling is encountered in the irradiated areas at this temperature (Figure S8, Supporting Information). All of these factors contribute to a more extensive surface coverage of facets than at 350 °C.

Regarding irradiation‐induced texture development at 550 °C, our previous study^[^ ^10^ ^]^ shows that irradiation at 550 °C leads to the development of a <100> texture (Figure S10, Supporting Information). Although these orientations facilitate channeling, dechanneling becomes inevitable due to increased mean square displacements at this temperature. The voids are primarily located within the grains, with a notable concentration of voids on the surface (refer to Figure 4), indicating the predominant role of the latter. These factors play a significant role in creating surface facets on nearly all grains in NC‐Ni irradiated at 550 °C.

## 4 Conclusions

Our work uncovers a novel mechanism for surface faceting. The formation of facets at various temperatures is attributed to surface stress induced by void swelling, with surface voids triggering the development of facets. Proximity of void distribution to surfaces was deemed necessary for facet formation, explaining the absence of facets or smooth grains where voids formed at greater distances. The crystallographic orientation strongly correlates with facet type. The <100> and <111> grains display smooth and wavy morphologies, while grains with orientations in between develop complex morphologies. The formation of different facets is directly linked to varying levels of stress and surface energies in grains of different orientations. Our findings clearly indicate that low values of both factors lead to the creation of wavy facets, while higher values result in the development of more intricate shapes.

Here, the development of surface facets and other related findings is demonstrated in NC‐Ni, a promising corrosion‐resistant coating for the primary and secondary structural materials of MSRs. Our recent investigations^[^ ^41^ ^]^ uncovered that impurities in the molten salts can induce the formation of corrosion‐induced surface facets, which, in turn, exacerbate corrosion rates in NC‐Ni. Although the phenomenon observed here is more unique to ion irradiation than actual neutron irradiated samples in MSR, the authors believe that the present results remain relevant for in situ ion irradiation–corrosion experiments. Several facilities employing both heavy ions and protons have been developed,^[^ ^42^, ^43^ ^]^ enabling simultaneous irradiation and corrosion to study the synergistic effects between the two processes. While currently, proton beams are used in simultaneous irradiation‐corrosion research to assess effects in FLiNaK corroding media, there is no fundamental limitation preventing high‐energy heavy ion facilities from conducting experiments in a similar medium. Moreover, these irradiations offer the advantage of achieving high dpa, relevant to MSR conditions. In such cases, void swelling and the related surface morphologies, such as those observed in the present study, could play a significant role in influencing corrosion behavior. These factors are crucial for accurately interpreting the corrosion behavior during in situ experiments.

## 5 Experimental Section

5.1

5.1.1

##### Sample Preparation

A Ti substrate was coated with an NC‐Ni coating (500 μm thick) with an average grain size of 20 nm using pulsed electrodeposition, as explained in detail in.^[^ ^33^, ^44^ ^]^ The resulting coatings were removed from the Ti substrate to create self‐standing NC‐Ni foils. Four samples with dimensions of 13 × 6 mm were cut from the as‐deposited NC‐Ni foils. These samples were mechanically polished using SiC grit (1200, 2000, 2400, 4000) and diamond (3 and 1 μm), followed by vibratory polishing using alumina with 0.06 μm.

##### Ion Irradiation

Irradiations were performed with 1.4 MeV Ni^+^ ions normally incident on the samples at temperatures of 250, 350, 450, and 550 °C using a 1.7 MV Tandetron accelerator at IGCAR. The reason for conducting irradiations at temperatures lower than the operating conditions of MSRs is to investigate phenomena within the thermal‐assisted regime, where both thermal and irradiation effects influence defect mobilities and consequently, microstructural evolution. At higher temperatures, these irradiation‐driven effects are largely masked by thermally dominated microstructural changes. The choice of this upper‐limit temperature (550 °C) also aligns with the peak swelling temperature for the dose rates employed in the present irradiations.

The NC‐Ni samples were placed on a heater stage, and the temperature during irradiation was measured using a thermocouple attached to the stage. During irradiation, a portion of the NC‐Ni was covered with a molybdenum mask. The area exposed to the beam is designated as the irradiated area, while the area beneath the mask, which experienced the same thermal history as the irradiated area but was shielded from the beam, is referred to as the unirradiated area. A raster scan with × and y frequencies of 1015 and 1036 Hz, respectively, was employed. The beam uniformity was verified using the fluorescence response of a quartz screen placed in the sample holder. Beam current measurements were performed by periodically inserting a Faraday cup positioned in front of the sample. During the sampling interval, the beam was blocked by the Faraday cup for a specified period (usually 20–30 s), enabling measurement of the average beam current. Following this, the Faraday cup was withdrawn to expose the sample to the beam for 900 s. The average current recorded in the prior sampling step was utilized to determine the incremental charge or dose delivered to the sample. This process was repeated continuously until the target fluence was reached. A beam current of 500 nA with a 5% variation was used for irradiations and the variation in temperature was ±1 °C.

During irradiation, the chamber vacuum was maintained at ≈5 × 10^−8^ mbar, and a cold finger was placed in close proximity to the sample to prevent contamination of the sample surface. The maximum displacement damage at the peak region was 18.5 dpa, and the corresponding fluence was 1.3 × 10^16^ ion cm^−2^. The displacement rate at the peak damage region was 3.8 × 10^−3^ dpa s^−1^. The damage profile was calculated using the damage energy method,^[^ ^45^ ^]^ which incorporates the energy absorbed and ionization energy loss of recoil particles obtained from SRIM full‐cascade simulations.^[^ ^46^ ^]^ A displacement threshold energy of 40 eV and a lattice binding energy of 5.9 eV,^[^ ^45^ ^]^ which is the sum of interstitial and vacancy formation energies in Ni, were employed in these calculations. Default values provided by SRIM were used for the surface binding energies. The damage profile and implanted Ni concentrations, simulated using SRIM, are shown in Figure S8 (Supporting Information).

##### Characterization

The surface topography of the irradiated and unirradiated areas of each sample was analyzed using a Zeiss Crossbeam 340 field emission SEM with an accelerating voltage of 5 kV. An AFM (M/s NT‐MDT) was used to investigate the variation in the surface morphology of the samples. The cross‐sectional TEM samples (lamella) were prepared using a focused ion beam (FIB) based lift‐out technique using a dual beam SEM Helios Nanolab 600i. Initially, the sample was polished using a 30 kV Ga^+^ beam at an angle of ±1.5° to the sample surface. Final thinning and cleaning were performed at 2 kV Ga^+^ with the angle of incidence of 6° for a duration of 5 min. The FIB‐prepared lamellae were examined using a 300 kV Titan Themis TEM microscope. The voids were identified by focus through sequence, such as under‐focus (Δ*f* = −1.6 μm) and over‐focus (Δ*f* = +1.6 μm) imaging conditions.

EBSD measurements were carried out on the irradiated area using a SEM (Zeiss‐Sigma‐300) coupled with an EBSD detector (Oxford Instruments) with an accelerating voltage of 20 kV. Scans were performed with a constant step size of 50 nm for the irradiated area. Kikuchi diffraction patterns generated during the process were captured by the complementary metal oxide semiconductor (CMOS) camera. Aztec and Aztec Flex software were used for data acquisition and analysis. GBs were identified with a misorientation greater than 5° between two adjacent pixels. Further, Brandon criteria were used for identifying Σ3 CSL boundaries with a tolerance angle of 8.66°.^[^ ^47^ ^]^

To understand the reason for facet formation, smooth and wavy facets with <001> and <111> orientations were identified using EBSD. A lift‐out was performed in this area (see Figure S11, Supporting Information), and the FIB lamella was attached to a TEM copper grid. This sample was then transferred to the TEM sample chamber for analysis.

##### Statistical Analysis

The experimental results presented here are derived from multiple measurements with a reasonable number of trials. More than 20 images of TEM and SEM were used for analysis, and representative figures are shown in the manuscript. In the case of EBSD, more than four scans were conducted for each sample at various locations within an area of 250 × 200 μm or larger to ensure statistical significance. Data acquisition and microstructural analysis were carried out using Aztec software. The orientation‐dependent morphology of surface facets was analyzed in over 100 grains.

## Supporting Information

Supporting Information is available from the Wiley Online Library or from the author.

## Conflict of Interest

The authors declare no conflict of interest.

## Supporting information

Supplementary Material

## Data Availability

The data that support the findings of this study are available within the manuscript and the Supporting Information. Other findings of this study are available from the corresponding author upon reasonable request.
