# Supplementary material for: Void Swelling Induced Surface Modifications: Exploring the Relation between the Crystallographic Orientation and Surface Facets
Source: Small Sci. 2026 Jan 13;6(1):e202500172. doi: 10.1002/smsc.202500172 (PMC12798783; doi:10.1002/smsc.202500172)
Supplement: Supplementary file 1 — Supplementary Material [file SMSC-6-e202500172-s001.pdf]

Supporting Information

Void swelling induced surface modifications: Exploring the relation between the crystallographic orientation and surface facets

S. Julie[1, 2], C. David[1, 2,*]

[1]*Indira Gandhi Centre for Atomic Research, A CI of Homi Bhabha National Institute (HBNI), Kalpakkam 603102, Tamilnadu, India.*

[2]*Materials Science Group, Indira Gandhi Centre for Atomic Research, Kalpakkam-603102, Tamilnadu, India*

**Corresponding Author, E-mail:david@igcar.gov.in*

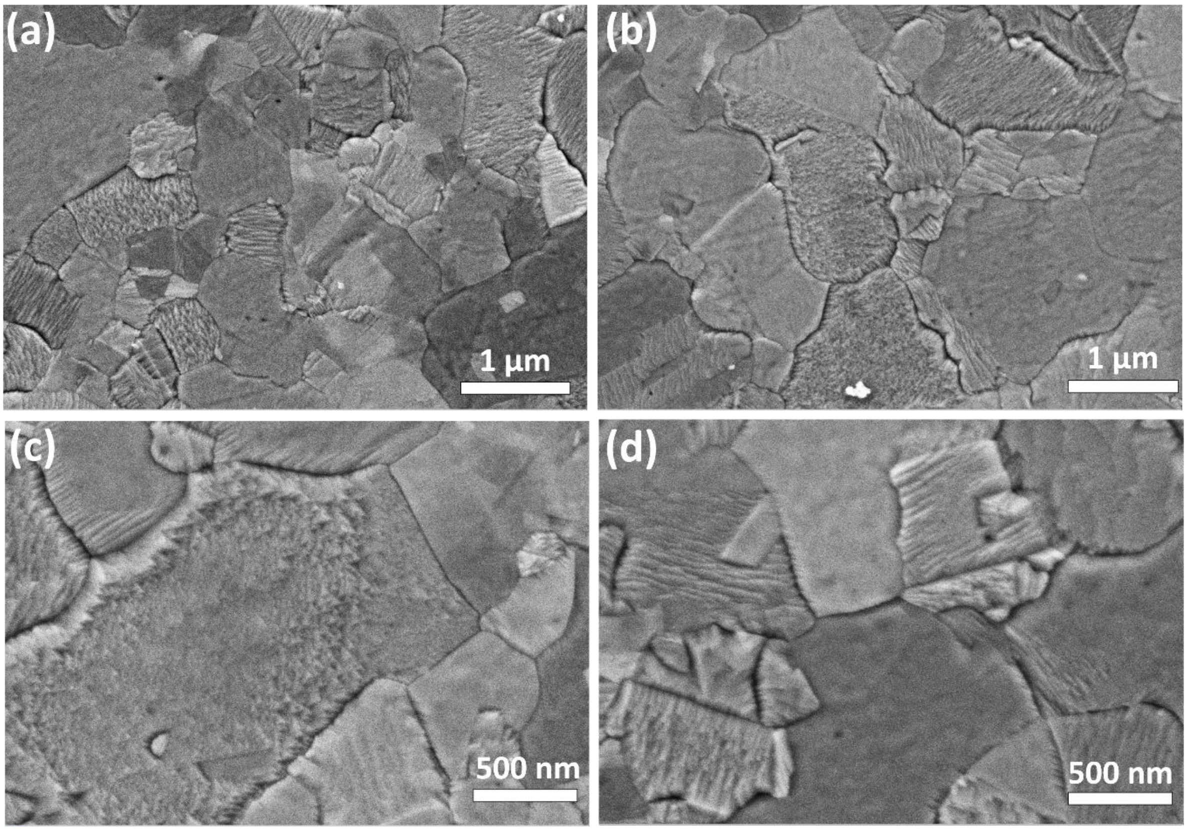

Figure S1: SEM image of facets formed during irradiation at 350°C. The majority of grains are devoid of facets. (a-d) Different types of facets at different magnifications.

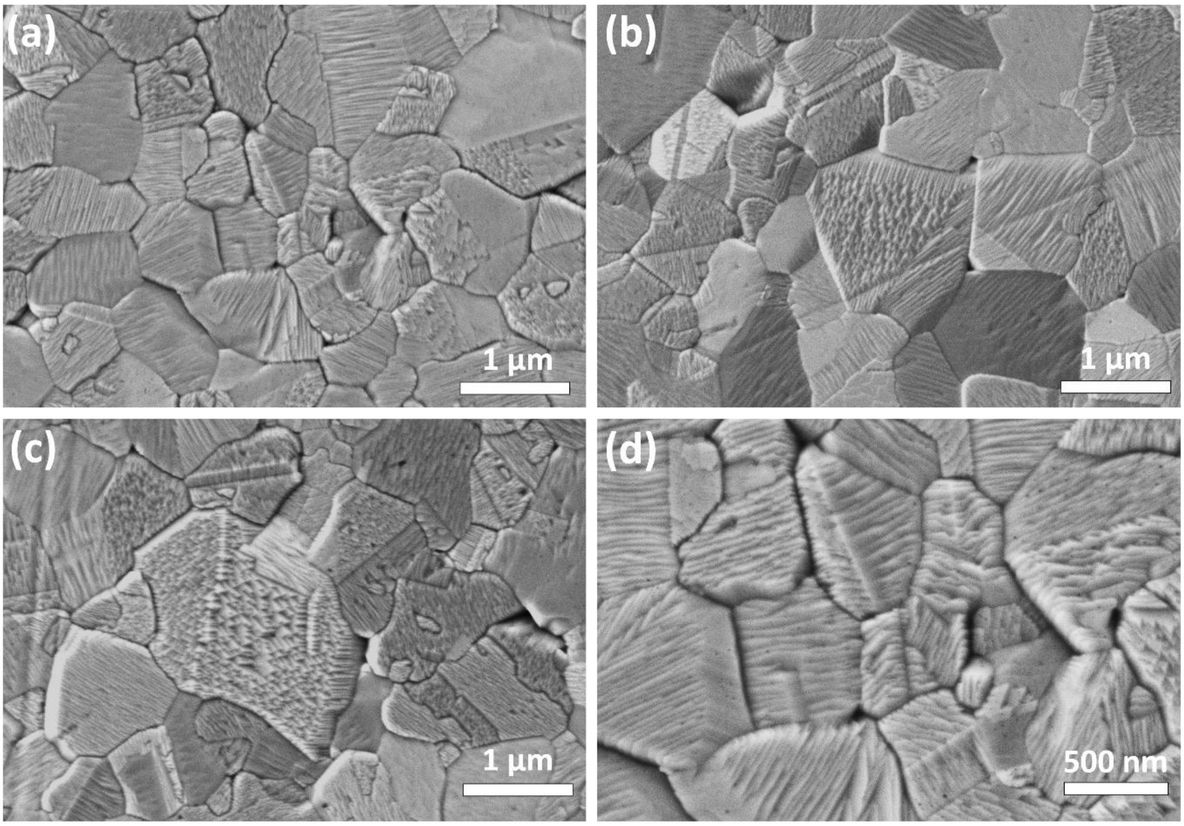

Figure S2: SEM image of facets formed during irradiation at 450°C. The majority of grains are faceted, and few grains appear as smooth. (a-d) Different types of facets at different magnifications.

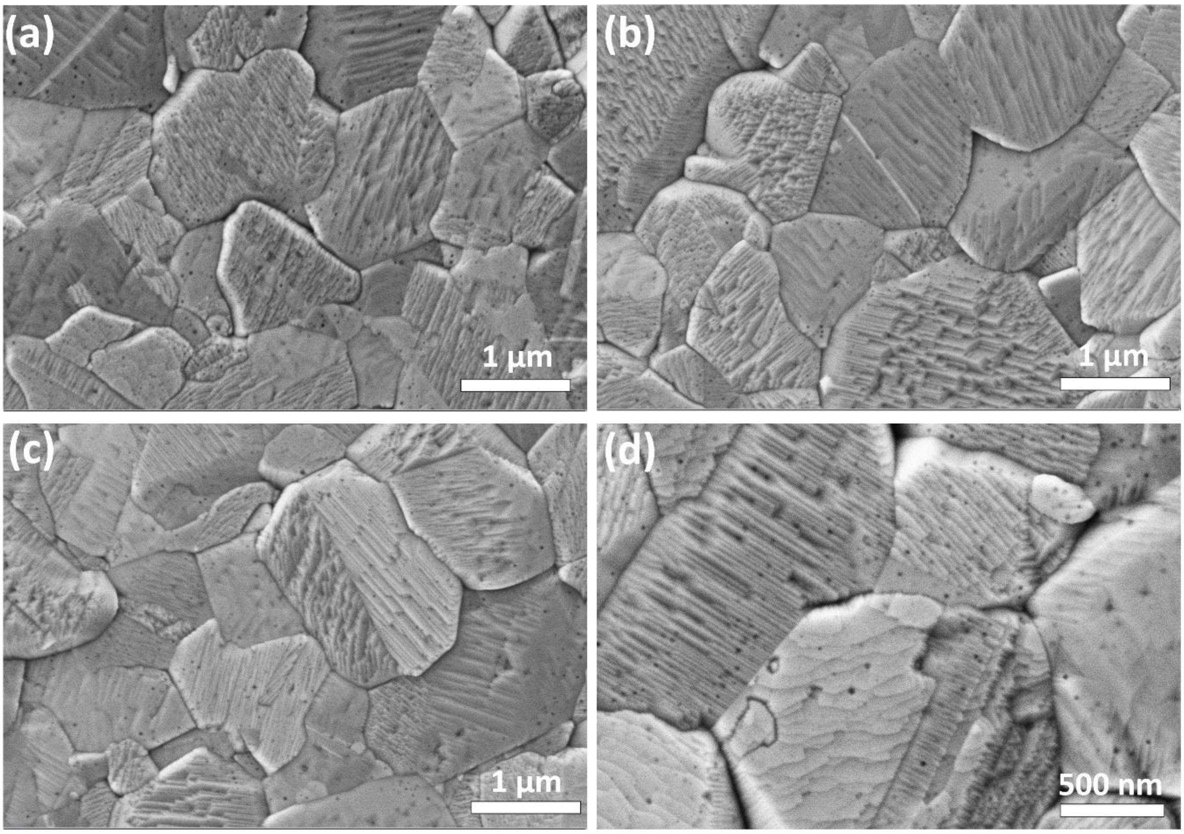

Figure S3: SEM image of facets formed during irradiation at 550°C. All grains are faceted. (a-d) Different types of facets at different magnifications.

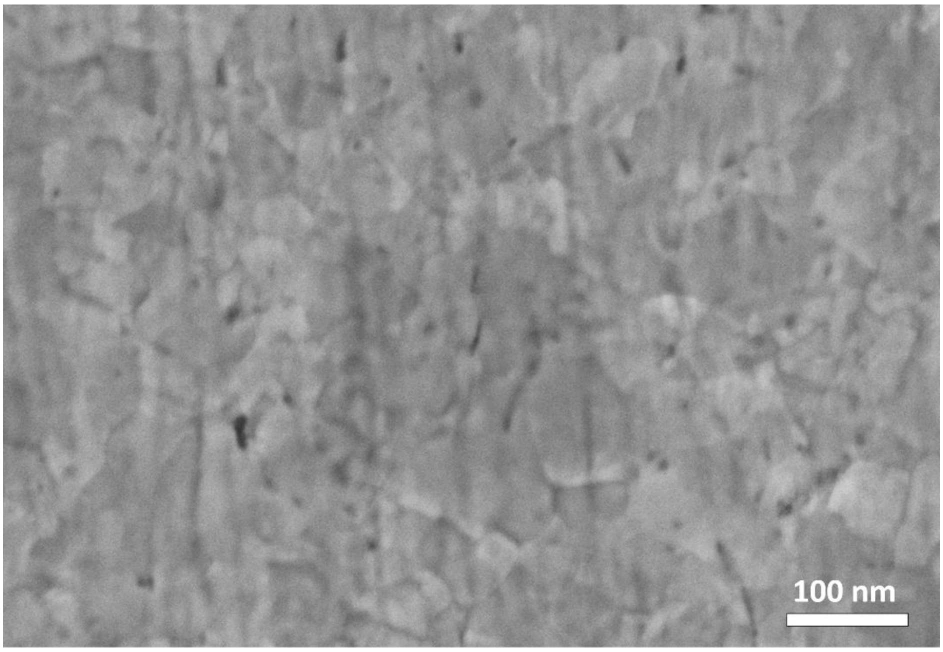

Figure S4: A panoramic SEM image of NC Ni surface irradiated at 250°C

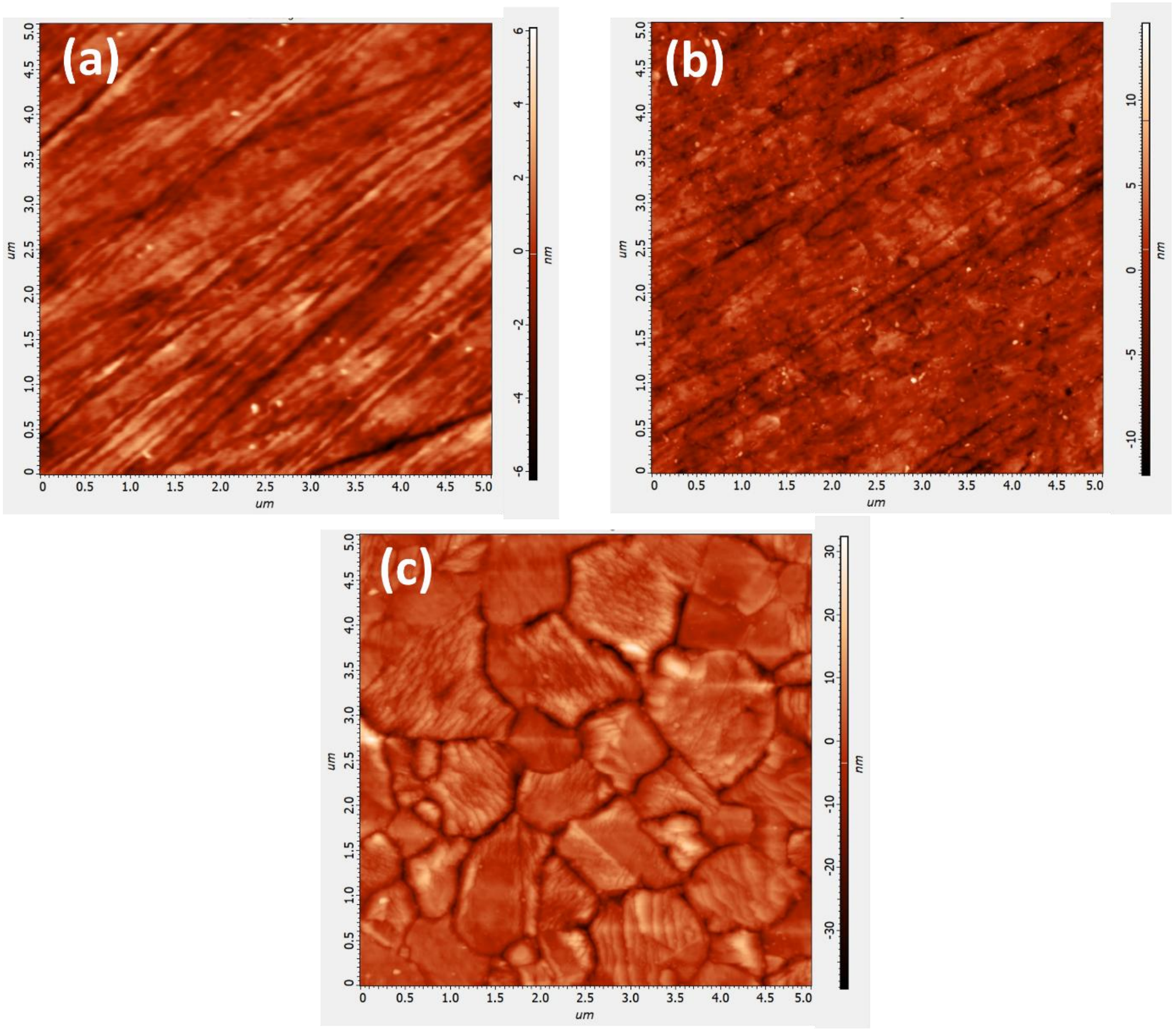

Figure S5: AFM images of NC Ni samples showing the surface morphology of (a) as-deposited (unirradiated), (b) irradiated at 250°C and (c) irradiated at 350°C.

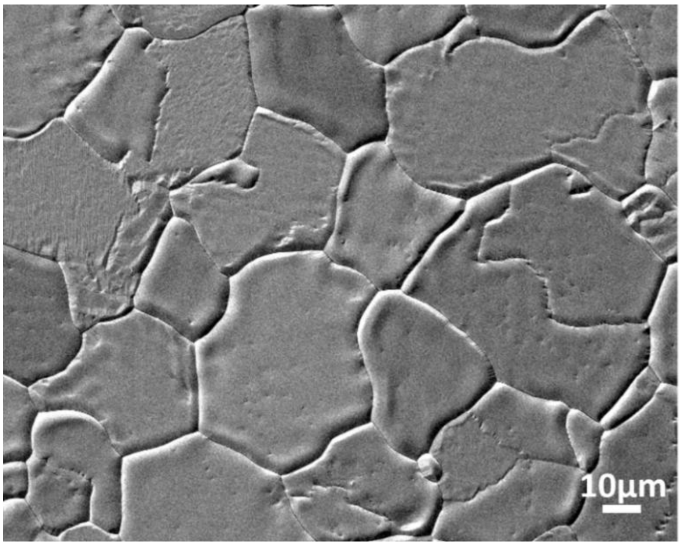

Figure S6: The SEM image of the NC Ni sample annealed at 650°C for 24 hrs. showing smooth grains devoid of surface morphological features.

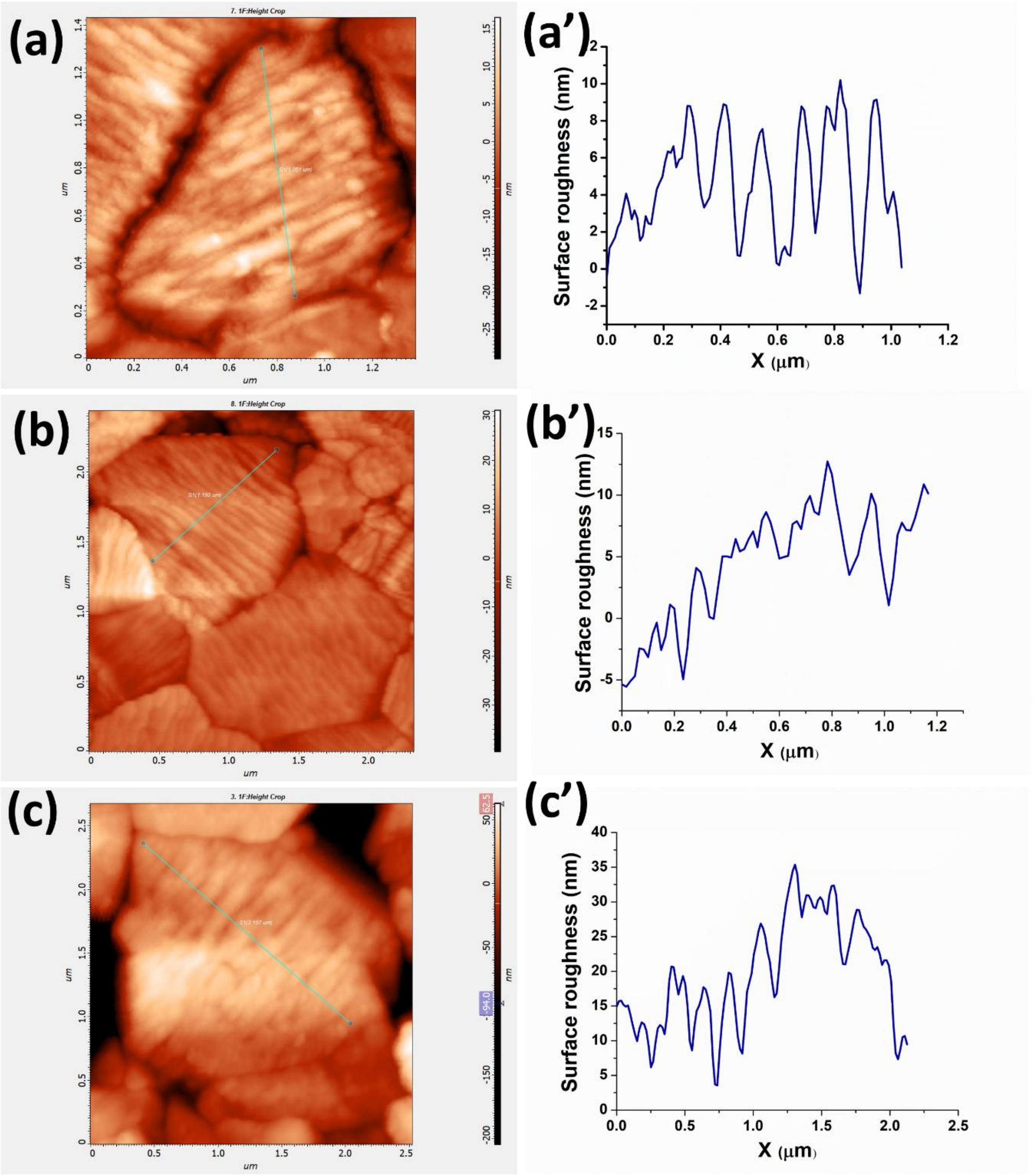

Figure S7: AFM images of NC Ni irradiated at different temperatures, (a) and (a') 350°C; (b) and (b') 450°C; (c) and (c') 550°C. (a), (b) and (c) are AFM 2D images of faceted surfaces and (a'), (b') and (c') are height profiles of lines marked across the faceted grains.

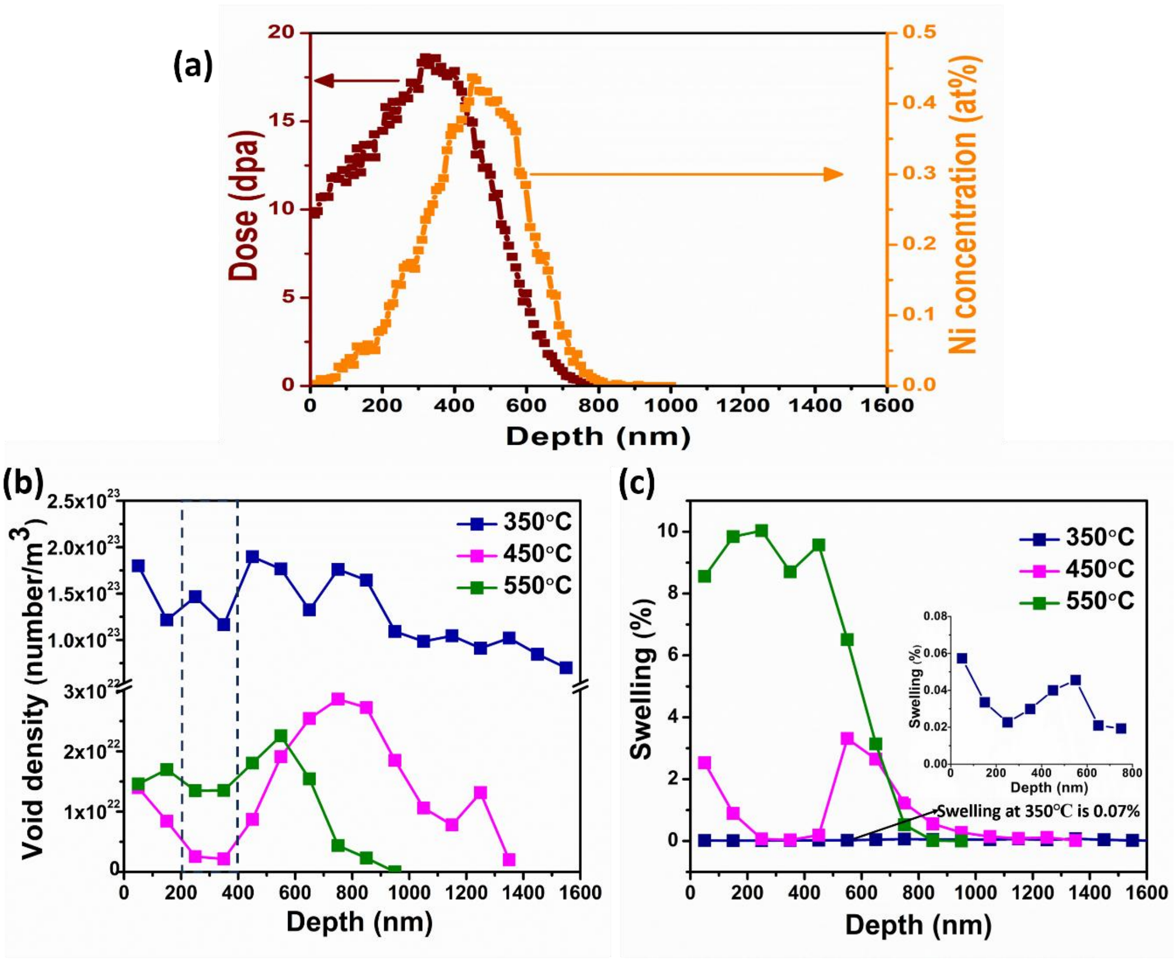

Figure S8: (a) The damage profile and the concentration of implanted nickel, as simulated using SRIM-2008. The depth profiles of (b) void density and, (c) void swelling in NC Ni irradiated at three temperatures. The injected interstitial effect is observed at depths between 200 nm and 400 nm (dotted rectangle). This effect is significant at 450°C and lesser at 550°C. However, the injected interstitial effect is absent at 350°C. The inset shows the enlarged view of void swelling at 350°C.

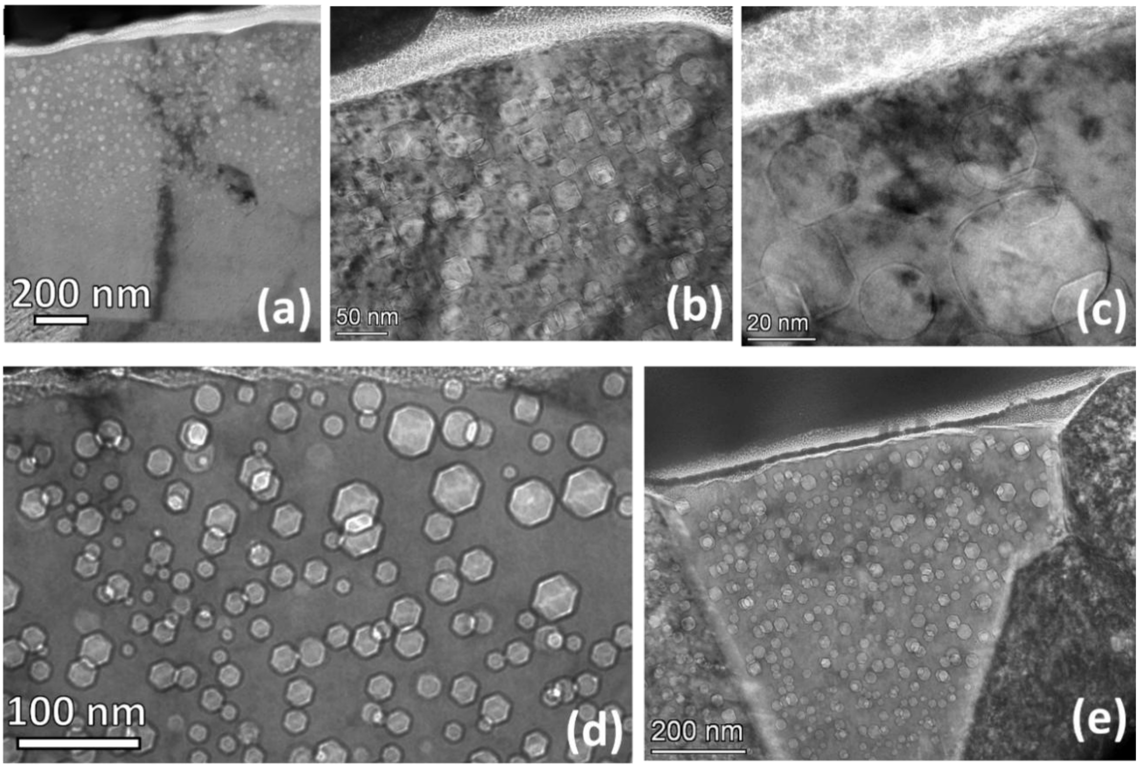

Figure S9: Voids formation and surface facets at 550°C. (a-c) Voids are formed near the surface, slightly smoother surface is visible, depicted clearly in (c). (d) and (e) Voids formed in close proximity to the surface, faceted surfaces are visible.

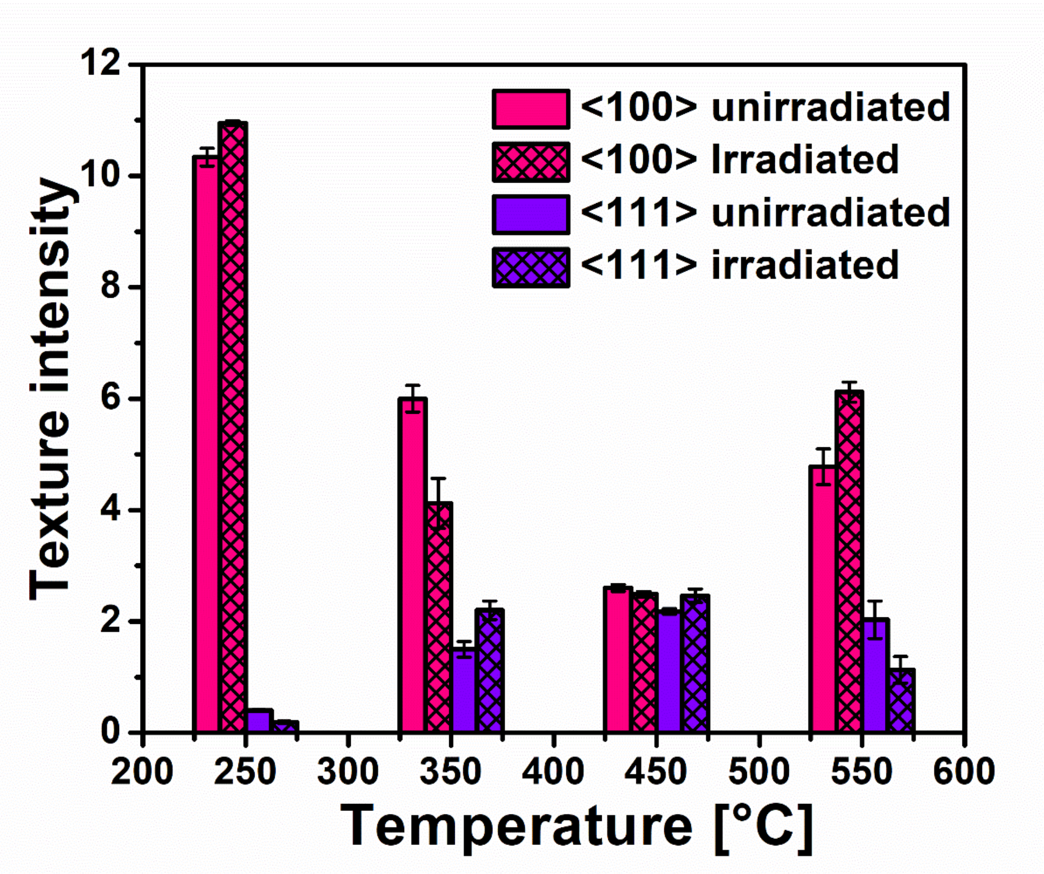

Figure S10: The texture intensity variation as a function of temperature for both unirradiated and irradiated areas. The texture intensities were calculated from the EBSD crystal orientation map and inverse pole figures. Readers can refer to Fig. 2 in [1] for more information.

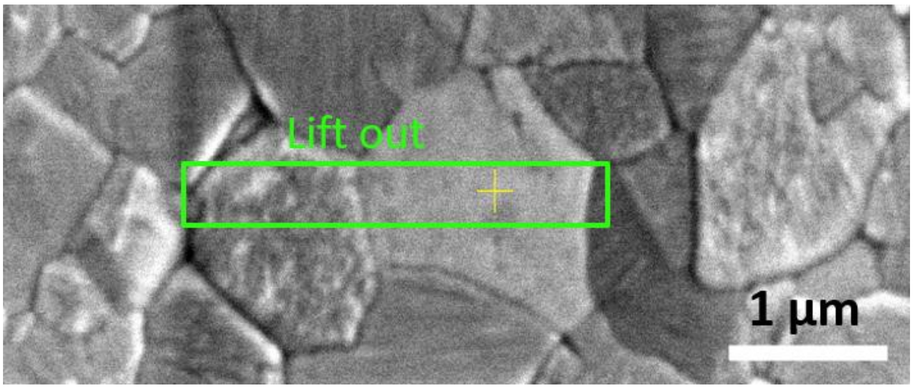

Figure S11: The FIB/SEM image of NC Ni irradiated at 450°C. The rectangular area shows the FIB region consisting of grain orientations <111> and <001>.

Calculation of critical angle

The critical angle Ψ_{uvw}^{c} is the angular width of channeling direction; at greater angles, ions undergo close encounters with atomic rows, increasing non-channeled fractions. The critical angle Ψ_{uvw}^{c} is expressed [2] as:

$$\Psi_{uvw}^{c} = \left[\frac{3a_{TF}^{2}Z_{1}Z_{2}\left(\frac{e^{2}}{4\pi\varepsilon_{0}}\right)}{Et_{uvw}^{3}}\right]^{1/4} \quad \textbf{(1)}$$

where, t_{uvw} is the atomic distance along the <uvw> direction, Z_1 and Z_2 are the atomic numbers of the projectile and target atoms (in the present case $Z_1=Z_2$), E is the energy of incident ion and a_{TF} is the Thomas-Fermi screening length and is calculated as:

$$a_{TF} = \frac{0.8853a_{0}}{\left(Z_{1}^{2/3} + Z_{2}^{2/3}\right)^{1/2}} \quad \textbf{(2)}$$

where a_0 is the Bohr radius (0.053nm).

Table S1 Estimated critical angle (Ψ_{uvw}^{c}) and non-channeling fractions (X_{uvw}) for Ni sample

**Direction**	$\boldsymbol{\Psi_{uvw}^{c}}$
<110>	3.76
<100>	2.89
<111>	1.91

References

[1] S. Julie, K. Mariappan, C. David, N.P. Wasekar, V. Shankar, A study on the competition and synergy between irradiation and temperature on the texture and recrystallization of nanocrystalline nickel, Appl. Surf. Sci. 638 (2023) 158085. https://doi.org/https://doi.org/10.1016/j.apsusc.2023.158085.

[2] B.W. Kempshall, S.M. Schwarz, B.I. Prenitzer, L.A. Giannuzzi, R.B. Irwin, F.A. Stevie, Ion channeling effects on the focused ion beam milling of Cu, J. Vac. Sci. Technol. B Microelectron. Nanom. Struct. 19 (2001) 749. https://doi.org/10.1116/1.1368670.

{
